# Therapeutic Alternatives in a Pediatric Patient With Molar Incisor Hypomineralization: A Clinical Case Report

**DOI:** 10.7759/cureus.88606

**Published:** 2025-07-23

**Authors:** Karla D de la Cruz-Fabián, Norma-Leticia Robles-Bermeo, Nayeli Lovera-Rojas, Carlo E Medina-Solís

**Affiliations:** 1 Advanced Studies and Research Center in Dentistry "Dr. Keisaburo Miyata" School of Dentistry, Autonomous University of State of Mexico, Toluca, MEX; 2 Academic Area of Dentistry of Health Sciences Institute, Autonomous University of Hidalgo State, Pachuca, MEX

**Keywords:** case report, children, dentistry, molar incisor hypomineralization, pediatric dentistry

## Abstract

A notable increase in the incidence of molar incisor hypomineralization (MIH) has been observed in recent years. This trend may be attributed to a combination of factors, including increased public awareness, improved diagnostic techniques, and a greater emphasis on early childhood oral health. Clinically, MIH initially presents as white-to-brown opacities without enamel loss, progressing to post-eruptive breakdown in severe cases, often accompanied by hypersensitivity and atypical caries that may lead to pulp involvement. MIH treatment requires a correct clinical assessment of the tooth as the first step toward selecting a therapy that will ensure long-term success for the patient. The choice of dental materials is a decision that the clinician must make based on scientific evidence, the manufacturer’s instructions, and the patient's general condition. The purpose of this case report was to present the clinical results of a 15-month follow-up of different therapy options in an 11-year-old male patient with MIH. The symptoms reported by the patient included hypersensitivity to hot and cold stimuli in the upper first permanent molars (FPMs) and tissue rupture in the lower FPM, which interfered with his eating function. The use of biomaterials, application of infiltrative resin (Icon®, DMG, Hamburg, Germany), preformed bands, topical applications of fluoride, as well as changes in oral hygiene habits and diet, were employed as therapeutics. The main contribution of this clinical case is that preserving the integrity of three molars with MIH in the same oral environment posed the greatest challenge to rehabilitation. However, with an individualized treatment plan, the evolution can be successful.

## Introduction

Molar incisor hypomineralization (MIH) is defined as a dental-enamel defect of systemic origin affecting at least one, and in some cases, all four first permanent molars (FPMs). It frequently encompasses the permanent incisors as well. The term was introduced by Weerheijm in 2003 and has since been adopted globally [1,2]. MIH occurs in 12.9%-14.2% of children and adolescents worldwide [2]; the United States has reported a prevalence of 9.6%, while, in Latin American countries such as Brazil, the figures rise to 13.48%. Studies in Mexico have revealed a prevalence of 15.8% in the general population [3-5].

The exact etiology of MIH remains uncertain; however, research suggests that disruptions during amelogenesis linked to prenatal factors (maternal stress, alcohol use, or birth complications) and childhood illnesses (fever, infections, respiratory conditions, or antibiotic use) may contribute to structural defects in permanent teeth [2,6]. Clinically, MIH manifests as demarcated opacities ranging from white-cream to yellow-brown, with severity-dependent progression. Advanced cases exhibit post-eruptive enamel breakdown, hypersensitivity, and functional impairment upon tooth eruption [1,2]. Mathu-Muju et al. [7] classified the levels of MIH according to the extent of compromised teeth.

Mild MIH: Delimited opacities in the FPMs, specifically in areas free of chewing stress. The opaque areas are isolated and exhibit no enamel fractures or caries. Only small, if any, hypomineralization lesions can be observed on the incisors. The patient has no history of dental hypersensitivity.

Moderate MIH: Atypical restorations can be observed. Opacities are present on the occlusal surfaces and in the incisal thirds, but without enamel ruptures. These may develop after tooth eruption, and caries lesions may ensue in one or two surfaces without, however, involving the cusps. The patient commonly reports tooth sensitivity.

Severe MIH: Enamel breakdown occurs during tooth eruption. The patient reports pain or sensitivity, and extensive caries lesions are frequently associated with the affected enamel. A broken crown may be observed with involvement of the pulp. There may be atypical restorations.

The developmental and qualitative enamel defects in MIH are caused by reduced mineralization, increased protein and water content, and altered inorganic enamel components, which can lead to discoloration, increased sensitivity, and post-eruptive breakdown of the affected teeth [8].

Restoring teeth affected by MIH poses significant clinical challenges due to their altered enamel structure. Beyond aesthetic concerns, particularly when the incisors are involved, these teeth frequently present with hypersensitivity and exposed dentin, increasing the risk of chronic pulp inflammation. The hypomineralized enamel's distinct composition further compromises restoration longevity, often leading to bond failure and recurrent marginal defects [1,2,9]. Treatment must consider factors such as age, severity of the defect, and associated symptoms. Clinicians should focus mainly on prevention and the early detection of hypomineralized teeth [9,10]. Initial therapeutic measures should include applying fluoride, controlling biofilm, using glass ionomer to seal or reconstruct defects, and/or implementing more advanced techniques such as the use of low-viscosity infiltrative resins. Diagnosis and treatment should be individualized for each dental organ, even when they belong to the same patient [2,9].

## Case presentation

An 11-year-old Mexican boy presented to the Pediatric Dentistry Specialty Clinic at the Autonomous University of the State of Mexico with his mother, who reported his chief complaint as a fractured molar causing chewing discomfort. The patient’s medical history revealed that his mother had a high-risk pregnancy (gravida 3, para 3) requiring bed rest during the first two trimesters due to threatened miscarriage, followed by a full-term but complicated delivery. No other significant medical history was noted. The mother stated that they chose this university clinic primarily due to its affordable treatment options.

**Figure 1:**
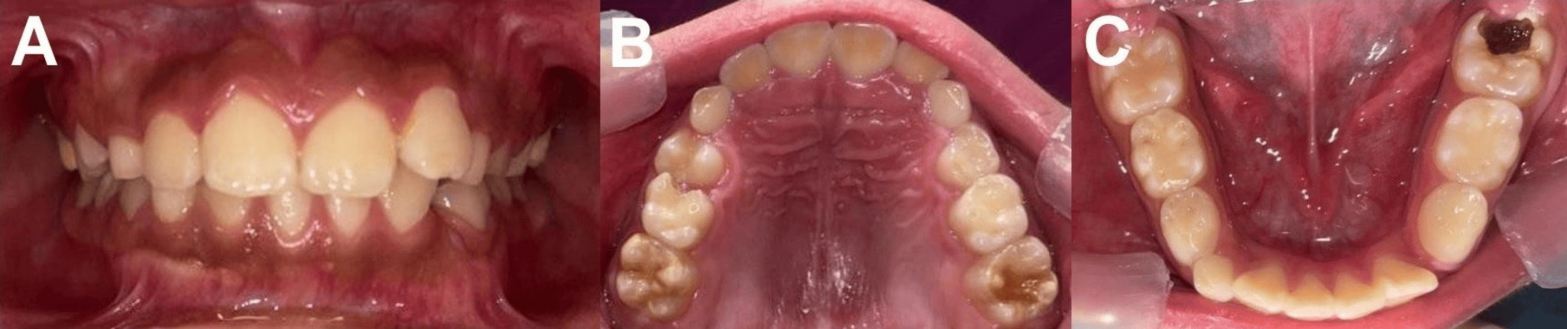
Initial photographs A) frontal bite, B) upper occlusal area, and C) lower occlusal surface

The patient reported symptoms that included hypersensitivity to hot and cold stimuli in the upper FPMs and tissue rupture in the lower FPM, which interfered with his ability to chew. A clinical examination revealed adequate oral hygiene, well-hydrated coral-pink gingival tissue, mixed dentition, and yellow-to-brown pigmentation on the occlusal surfaces of the upper FPMs, as well as caries in the mesial of the second temporary molar and the lower left FPM (Figures 1A-1C).

We diagnosed MIH according to Mathu-Muju et al. [7], and recorded the patient’s caries experience using the Decayed, Missing, and Filled Primary Teeth (DMFT) Index. The caries lesions were graded according to the criteria established by the International Caries Detection and Assessment System (ICDAS), and DIAGNOdent™ Pen 2190 (KaVo, Biberach, Germany) was employed to measure the MIH-induced loss of mineral content in the enamel. Our assessment included digital photographs, which were taken under the mother's written consent. Evaluation and treatment were conducted by a single examiner, with calibrations determined according to the DMFT and ICDAS indices.

The patient presented the following risk factors: a diet high in sugar and carbohydrates, and a pattern of short intervals between meals, which, according to the Stephan curve, consistently alter oral pH. Based on the above clinical characteristics, we diagnosed MIH and focused treatment not only on preserving or restoring the compromised structure of his FPMs but also on mitigating his hypersensitivity.

We observed an ICDAS 6 caries lesion in the lower left FPM. Ensuring full isolation, we administered local anesthesia and applied a rubber dam, using a lower alveolar block technique with 2% lidocaine containing 1:100,000 epinephrine, before removing the lesion with high-speed ball burs and manual instruments. The patient initially exhibited sensitivity, necessitating a supplemental dose of anesthetic, which successfully alleviated the discomfort.

**Figure 2:**
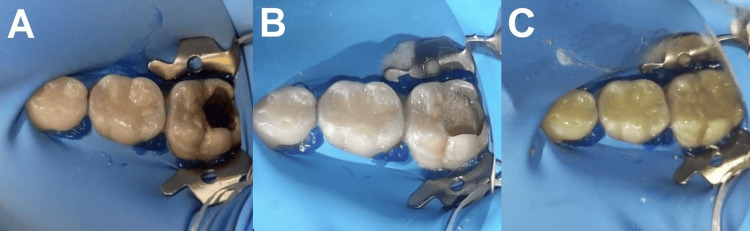
A) Initial photograph of ICDAS 6 lesion, B) caries-free cavity and placement of Biodentine®, and C) final restoration with glass ionomer

Behavior management techniques appropriate for the patient's age were employed, and the patient displayed adequate cooperation throughout the treatment process. Due to the depth of the cavity, we placed Biodentine® (Septodont, Saint-Maur-des-Fossés, France) in the orifice and used a glass ionomer (EQUIA® Forte, GC Corporation, Tokyo, Japan) as a filling to reconstruct the occlusal surface. Considering the significant loss of structure and the depth of the lesion, we selected biomaterials with the highest likelihood of preserving the health of the pulp and tissue structure (Figures 2A-2C).

**Figure 3:**
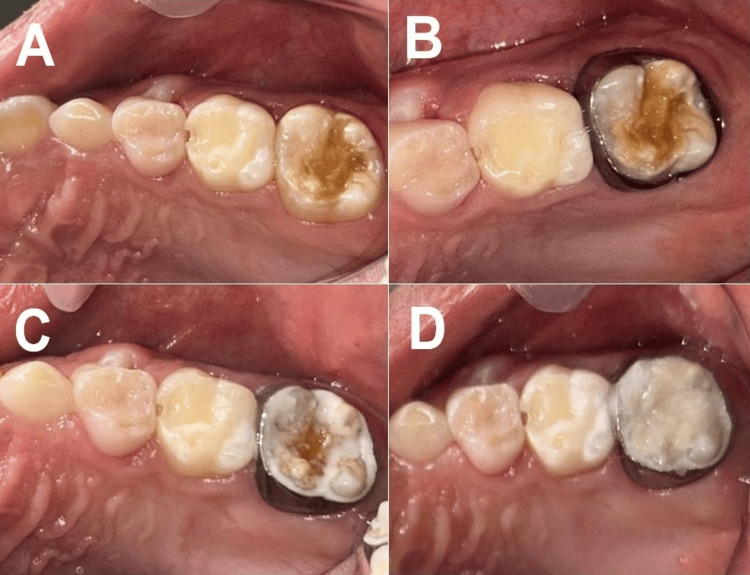
A) Initial photograph, B) band test, C) cemented band, and D) reconstruction with ionomer

As the upper left FPM exhibited moderate MIH with post-eruptive rupture compromising the structure of the crown, we placed a preformed band in the affected area to protect the remaining tissue. After adjusting and cementing the band with Gold Label I glass ionomer (GC Corporation, Tokyo, Japan), we reconstructed the occlusal surface of the molar with high-viscosity ionomer EQUIA® Forte (GC Corporation, Tokyo, Japan) (Figures 3A-3D). The preformed orthodontic band was selected to reinforce the structurally compromised molar, providing circumferential support to prevent enamel fractures while maintaining occlusal function. This approach is particularly useful in severe MIH cases where conventional restorations may fail due to poor enamel bonding [11,12].

As previously mentioned, we examined the upper right FPM using DIAGNOdent™ Pen 2190 to detect and monitor the initial caries lesions. Through the emission of laser light, this device illuminates decalcified areas of the tooth. By generating fluorescence, it helps determine the severity of demineralization by means of an acoustic signal and a numerical value. In the present case, we obtained a value of 37 for the occlusal and smooth surfaces, indicating intense demineralization according to the manufacturer's table of values (Table 1).

**Table 1:** Fissure caries and smooth surface caries

Type of caries	Fissure caries	Proximal caries
Measured value	0–12: Healthy tooth substance	0–7: Healthy tooth substance
Measured value	13–24: Initial demineralization	8–15: Initial demineralization
Measured value	>25: Strong demineralization	>16: Strong demineralization

**Figure 4:**
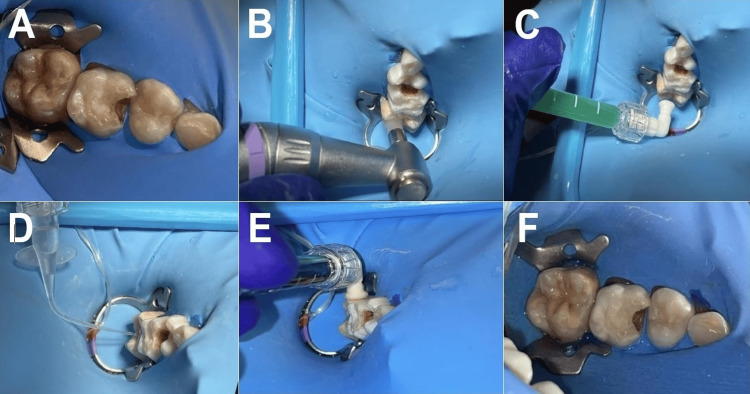
A) Initial photograph, B) prophylaxis, C) hydrochloric-acid placement, D) icon-dry, E) infiltrative resin application, and F) final photograph

The above grading corroborated the high porosity of the affected tissue because of deficient mineralization. In similar cases, clinicians have sought alternatives to replace the lost components, achieving favorable outcomes via infiltrative resins such as Icon® (DMG, Hamburg, Germany), particularly where no enamel ruptures have occurred. The manufacturer's detailed protocol for Icon® (DMG, Hamburg, Germany) was followed. We applied this material to the patient’s upper right FPM. We blocked sensitivity with local anesthesia, ensured full isolation, and performed prophylaxis of the area using hydrogen peroxide to eliminate food remains. We injected 15% hydrochloric acid (Icon-Etch, DMG, Hamburg, Germany) for two minutes, then rinsed the area thoroughly with abundant water. We added air from the triple syringe, followed by ethanol (99%; Icon-Dry, DMG) on the surface. We repeated this protocol three times until a decrease in the tissue discoloration was observed. Subsequently, we applied the infiltrative resin (Icon®, DMG, Hamburg, Germany), rubbing the area for three minutes with the unit’s light lamp turned off. We then carried out three applications and performed photocuring for 40 seconds on each surface. Finally, we removed the rubber dam and concluded by evaluating the procedure (Figures 4A-4F).

Among the general therapeutic measures taken to help the patient, we performed prophylaxis and applied 3M™ Clinpro™ White Varnish (3M Espe, Saint Paul, MN, USA) with 5% sodium fluoride to both arches. We also provided the patient and his mother with instructions on the correct brushing technique and emphasized the importance of following a diet low in sugar and fat.

**Figure 5:**
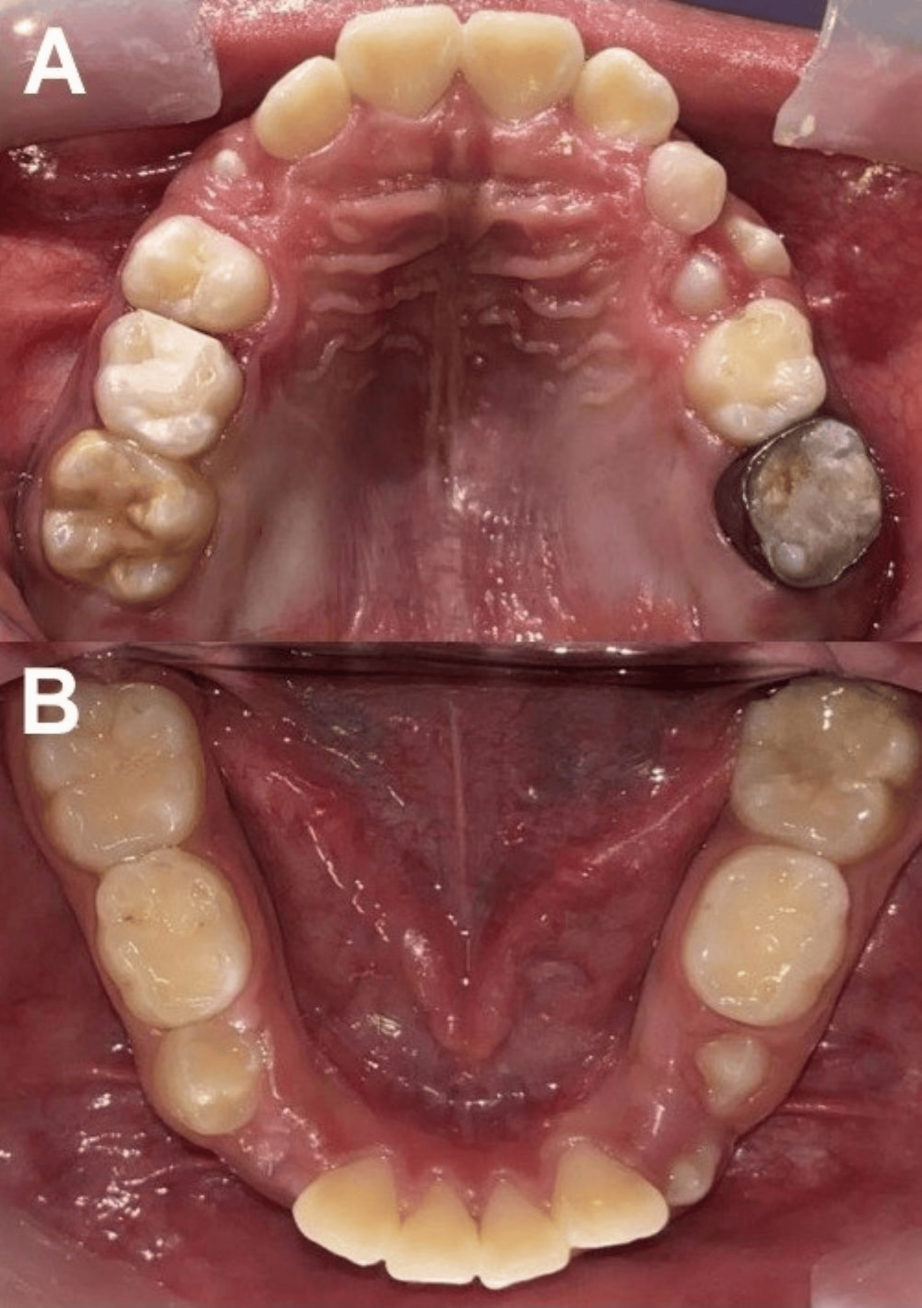
Photographs at a six-month follow-up A) Upper occlusal area and B) lower occlusal area

The patient attended a follow-up appointment after six months. During the visit, we noted that his hygiene was adequate, the color of the soft tissues was healthy, the previous treatments remained in excellent condition, and the patient reported no discomfort while eating or during daily activities. The hypersensitivity in his upper molars had subsided. Once again, we performed prophylaxis, applied fluoride, and reinforced oral hygiene instructions. A subsequent appointment was set to continue monitoring the health status of the patient (Figures 5A-5B).

**Figure 6:**
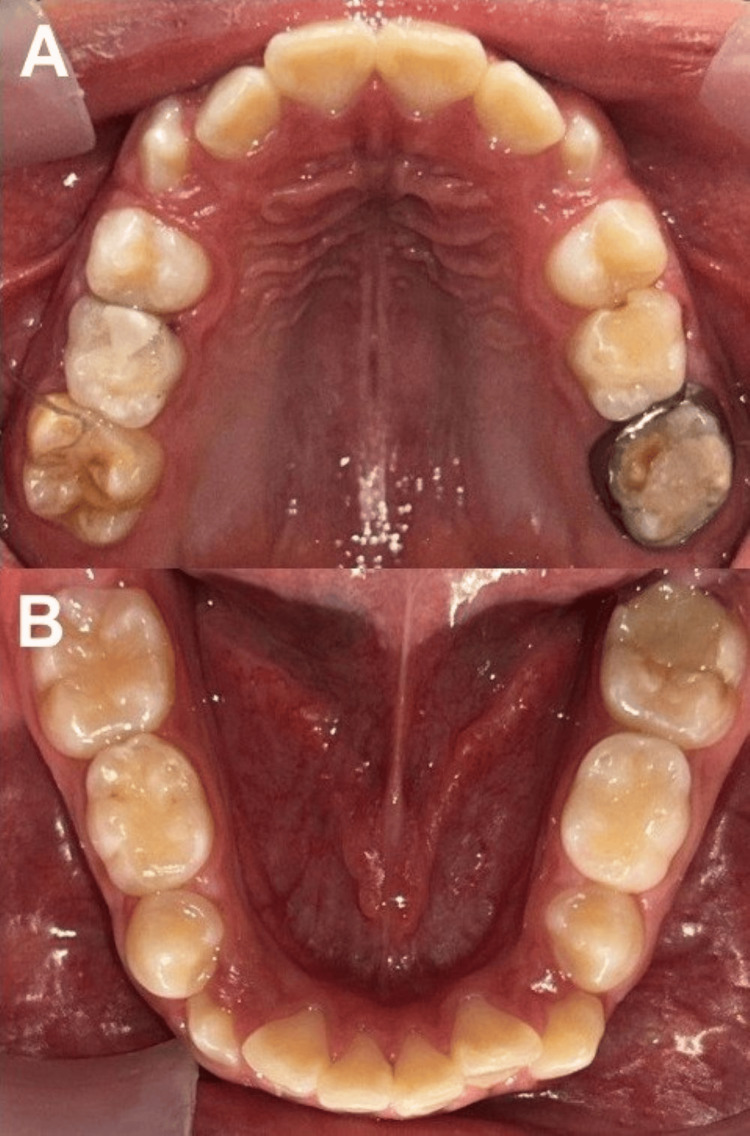
Photographs at a 10-month follow-up A) Upper occlusal view and B) lower occlusal view

At 10 months post-treatment, the patient continued to present adequate oral hygiene, and the treated molars had maintained their structure. Hypersensitivity was minimal. He reported being able to brush his teeth without discomfort and not needing to change his diet because of painful symptoms. During the appointment, we again reinforced oral hygiene measures and once again highlighted the importance of eating a low-sugar diet (Figures 6A-6B).

**Figure 7:**
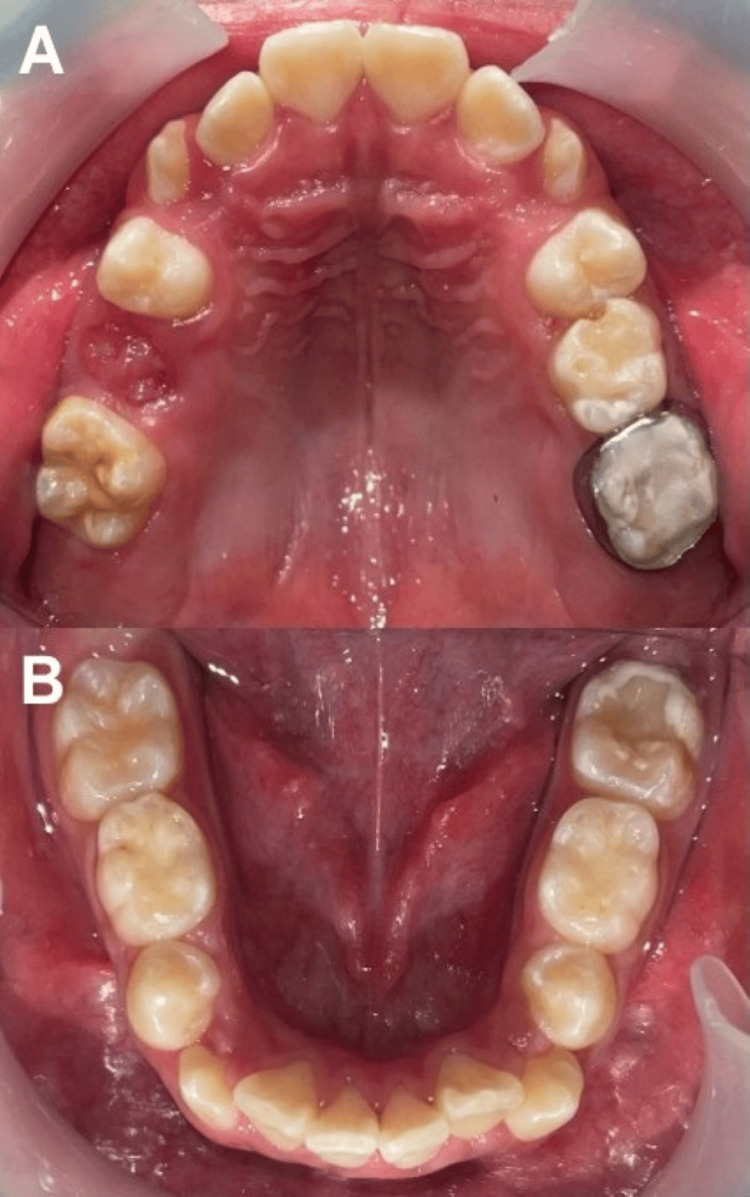
Photographs at a 15-month follow-up A) Upper occlusal area and B) lower occlusal area

At 15-month follow-up, we performed a clinical examination of the MIH-affected FPMs and noted the following: the tissue damaged by hypomineralization was preserved, the life span of the dental organs was extended, and the interval before requiring more invasive treatment had been prolonged, necessitating only minimal intervention. We measured the status of the MIH-affected tissue using a DIAGNOdent™ Pen 2190 and obtained scores ranging from 0 to 13 for the FPMs restored with ionomer. According to the manufacturer’s reference table, these values fall within a healthy parameter; this was achieved by mineralizing the structure and applying fluoride, both of which are recommended for successful long-term maintenance. To attenuate the patient’s hypersensitivity, we prescribed MI Paste Plus™ for FPM 26. This material contains fluoride and RECALDENT™ (CPP-ACP), a milk-derived protein recognized for its unique ability to deliver bioavailable calcium and phosphate (Figures 7A-7B).

Owing to the vast variety of clinical manifestations and symptoms of MIH, work continues to establish standardized treatment protocols capable of ensuring the conservation of tissue from hypomineralized teeth. In this case, a combination of therapeutic approaches successfully prevented structural deterioration of the patient’s FPMs over 15 months, avoiding the need for restoration, root canal therapy, or, in the worst case, extraction (Table 2).

**Table 2:** Therapeutic procedures performed in the case Among the general therapeutic measures taken to help the patient, we performed prophylaxis and applied 3M™ Clinpro™ White Varnish with 5% Sodium Fluoride on both arches. We also provided him and his mother with instructions on the correct brushing technique and emphasized the importance of following a diet low in sugar and fats.

Tooth	Treatment
Lower left first molar	Biodentine® (Septodont, Saint-Maur-des-Fossés, France); glass ionomer (EQUIA® FORTE, GC, Japan)
Upper left first molar	Metallic band with GOLD LABEL I glass ionomer (GC); reconstruction of the molar´s occlusal surface with high-viscosity ionomer (EQUIA® FORTE, GC, Japan)
Upper right first molar	Use of infiltrative resin (Icon®; DMG, Hamburg, Germany)
Lower right first molar	Use of 3M™ Clinpro™ White Varnish (3M Espe, Saint Paul, MN, USA) with 5% sodium fluoride

Written informed consent was obtained from the patient’s mother for the publication of this case report and disclosure of images. The patient’s data were handled with full confidentiality, and the study was conducted per the Code of Ethics of the World Medical Association Declaration of Helsinki. Patient care adhered to the Mexican Official Standard 013 (NOM-013-SSA2-2015), which adheres to clinical care under authorization of informed consent and patient assent. All interventions were only performed after written informed consent from the patient’s mother and assent from the patient.

## Discussion

The term “molar incisor hypomineralization” (MIH) was coined by Weerheijm et al. in 2001 and was promptly adopted by professionals around the world, necessitating a system to classify this condition [1]. Mathu-Muju et al. graded hypomineralization as mild, moderate, or severe according to the color and size of the associated opacities, extent of lost tissue structure, presence of caries, atypical restorations, and degree of hypersensitivity [7]. In Mexico, MIH cases have been classified as moderate in 67.1%, mild in 18.5%, and severe in 14.4% of cases [10]. The patient analyzed in this manuscript exhibited both moderate and severe instances of hypomineralization, characterized by opaque areas on occlusal surfaces, post-eruptive ruptures, and hypersensitivity.

The literature presents a variety of causes of MIH; however, in the last 10 years, numerous etiological hypotheses have focused on the pre-, peri-, and postnatal periods. This focus relates to the fact that, from the end of pregnancy until three years of age, children may experience alterations during the maturation of ameloblasts which modify their function [2]. Juárez-López et al. found that a history of maternal alcohol consumption, complications during labor and/or birth, the presence of infections, fever, stress, respiratory problems, and use of antibiotics, which affect the amelogenesis process, contribute to permanent structural defects in dentition [6]. In our case, we documented a history of maternal complications during pregnancy emerging as a likely risk factor for MIH. Specifically, the mother was prescribed relative bed rest during the first two trimesters owing to a threat of involuntary miscarriage and low weight. It can reasonably be assumed that these conditions were associated with the occurrence of MIH in our patient. However, the mother reported no issues with the child’s primary teeth, but we could be facing a memory bias, as in his permanent teeth, she noticed the problem with the child’s molars when he was 10 years of age, and they went to the dentist when the child was 11 years old.

Once the etiology of MIH is established, clinicians should recognize that enamel defects impair people's quality of life, entailing aesthetic and functional modification of the teeth [13]. Freitas et al. reported a correlation between MIH and reduced quality of life of patients and the presence of MIH; they found that it causes hypersensitivity, making it difficult for patients to brush their teeth and consume food, as well as hot or cold drinks. However, it also involves post-eruptive breakdown, and caries were identified as consequences that most affect patients [14]. In this case, the patient developed post-eruptive ruptures and reported hypersensitivity when brushing his teeth and consuming food or cold drinks. These symptoms impacted his quality of life but improved significantly following rehabilitative treatment.

Recent years have witnessed a surge in the prevalence of MIH, requiring increased efforts to find treatment strategies capable of preventing the premature loss of teeth attributable to this condition and improving patients’ quality of life [2,9,13]. Our patient presented with varying degrees of MIH, ranging from moderate to severe. When present, this variation constitutes a key element in treatment decisions, highlighting the fact that even if teeth are located in the same oral environment, the prognosis for individual teeth can differ, and these differences are influenced by several factors. These include appropriate choice of restorative material, use of preventive measures, cooperation from patients and parents, and consistent follow-up. The importance of these elements was underscored by the European Academy of Pediatric Dentistry, which highlights the need for early implementation of a tailored, evidence-based preventive strategy [2,6,15].

The implementation of different therapies is based on scientific evidence. Durmuş et al., among others, have used glass ionomer on molars to compensate for tissue loss [16]. Because of its properties, this material appears to offer effective mechanical resistance, especially when paired with fluoride varnish. In our case, there was not enough healthy tooth structure to support the walls of tooth 26; therefore, we placed a preformed orthodontic band to reinforce the resistance of the ionomer, this avoiding fractures. Glass ionomer is one of the most recommended materials for the intermediate treatment of molars affected by MIH due to its favorable properties, especially the one modified with resin, which seems to offer better mechanical resistance. However, its use in extensive restorations on posterior teeth makes them prone to fracture, so the joint use of a preformed orthodontic band provides greater support and protection of the affected tissue, helps prevent fractures, and is an economical option [11]. Bagattoni et al. utilized this technique with optimal results in a six-year-old patient. A follow-up at 36 months was carried out, ensuring success [12].

A variety of treatment options are available for molars with severe MIH. Biomaterials such as Biodentine®, made from calcium silicate, have proved to be highly compatible with dental tissues, promoting the formation of secondary dentin, as demonstrated by Ghilotti et al. [17]. Their findings corroborated the benefits of using this biomaterial for direct or indirect pulp coverage. We chose Biodentine® for our patient, given that his lower left FPM presented post-eruptive rupture and a deep caries lesion; this material allowed us to preserve the pulp in optimal condition and ensured the absence of postoperative sensitivity.

In an attempt to minimize the physical damage resulting from mild MIH lesions, Nogueira et al. [18] implemented low-viscosity resin infiltration versus the effects of fluoride applications in 51 patients over a period of 18 months. This approach, using ICON infiltrative resin®, achieved excellent results, promoting the obliteration of porosities and preventing the progression of the lesion. This, in turn, enhanced the structural integrity of the affected teeth by reducing the risk of enamel breakdown. We used this product for the same purpose with our patient. At 15-month follow-up, the FPM continued to exhibit marked opacities, but no loss of structural integrity had occurred, and hypersensitivity had decreased considerably, confirming that use of this material constitutes an effective technique with a high probability of success in the treatment of MIH.

The high level of porosity and mineral loss in the enamel resulting from MIH prompted us to search for a tool that could measure the quantitative value of tissue before and after applying the selected therapeutics. We opted for the DIAGNOdent™ Pen 2190 because it measures the fluorescence emitted upon contact of the enamel with light from a laser. Based on this, it assigns fissure caries and smooth surface caries as follows: “healthy tooth substance” values of 0-12, “incipient demineralization” values from 13-24, and “intense demineralization” values with a range >25 [19].

Altan et al. studied the use of DIAGNOdent™ Pen 2190 in MIH, testing the effectiveness of its application on infiltrative resins in 37 patients, and achieved final values indicating “healthy tooth substance.” This finding confirms the fact that, although the DIAGNOdent™ Pen 2190 does not remineralize teeth, it does provide structural filling of enamel porosities, resulting in reduced fluorescence at the time of measurement [20]. In the present case, initial DIAGNOdent values of 28-37 in the upper right FPM decreased to 0-13 after the use of Icon® (DMG, Hamburg, Germany) infiltrative resin, indicating structurally healthy tissue. The importance of these figures lies in the fact that the use of infiltrative resins, along with glass ionomer and fluoride, proved effective in reducing the fluorescence captured by the DIAGNOdent™ Pen 2190.

In this case, minimally invasive therapies were implemented for case management, considering the degree of involvement of the FPMs and their symptoms. In addition, the patient was advised to use MI Paste Plus. At follow-up appointments, fluoride varnish applications were performed, as recommended by the European Academy of Paediatric Dentistry policy document, which describes the use of pastes with hydroxyapatite and tricalcium phosphate due to their high success rate in treating demineralization and hypersensitivity [2].

Minimally invasive therapies are currently employed to preserve the structure of teeth affected by MIH [2,9,15]. In the present case, the pursuit of a favorable prognosis led us to adopt a combination of treatment approaches, such as fluoride application, biofilm control, high-viscosity glass ionomer restoration, the use of biomaterials for pulp protection, preformed bands, and resin infiltration. These interventions were adapted to meet the patient's clinical needs. Follow-up results confirmed that simple and minimally invasive treatments can provide positive clinical and psychosocial outcomes.

One limitation of this case report is that long-term treatment success depends on patient adherence to the recommended oral hygiene practices. While the patient and his parents followed the prescribed regimen during treatment, maintaining these practices post-treatment completion is crucial. Additionally, it is important to note that the positive outcomes observed in this case may not be universally applicable. Each patient with MIH requires an individualized treatment plan tailored to their specific needs. Nevertheless, this case contributes to the body of knowledge by demonstrating various therapeutic options for managing MIH.

## Conclusions

The case analyzed in this report was particularly complex, inasmuch as different stages of MIH had developed in the same oral environment. This required an individualized treatment for each FPM, ranging from minimal invasion to restoration. Raising awareness among patients and their parents about MIH is essential: given that it is a non-preventable pathology, the management of clinical manifestations requires mutual cooperation. At the 15-month follow-up, the dental status of our patient was stable, his FPM had no loss of structure, and sensitivity had diminished. This outcome was achieved through the use of bioactive materials and an approach aimed at halting the disease’s progression, which could otherwise lead to destruction of the tooth. Much remains to be learned about MIH; however, the aim of any treatment should be to improve the quality of life of the patient. The presented clinical case highlights the importance of a multidisciplinary approach to the management of MIH. The long-term follow-up of this patient underscores the potential for successful treatment outcomes.
